# New Cucurbitane Triterpenoids and Steroidal Glycoside from *Momordica charantia*

**DOI:** 10.3390/molecules14124804

**Published:** 2009-11-25

**Authors:** Jie-Qing Liu, Jian-Chao Chen, Cui-Fang Wang, Ming-Hua Qiu

**Affiliations:** State Key Laboratory of Phytochemistry and Plant Resources in West China, Kunming Institute of Botany, Chinese Academy of Science, Kunming 650204 Yunnan, China; E-Mails: jqliu18@163.com(J.Q.L.); jcchen@mail.kib.ac.cn(J.C.C.); wlycf@163.com(C.F.W.)

**Keywords:** *Momordica charantia*, cucurbitane triterpenoids, steroidal glycoside, cucurbitaceae

## Abstract

Three new cucurbitane triterpenoids **1**–**3** and one new steroidal glycoside **4**, were isolated together with ten known compounds from *Momordica charantia*. The structures of new compounds were determined to be 19(*R*)-*n*-butanoxy-5*β*,19-epoxycucurbita-6,23-diene-3*β*,25-diol 3-*O*-*β*-glucopyranoside (**1**), 23-*O*-*β*-allopyranosyle-cucurbita-5,24-dien-7*α*,3*β*,22(*R*),23(*S*)-tetraol 3-*O*-*β*-allopyranoside. (**2**), 23(*R*),24(*S*),25-trihydroxycucurbit-5-ene 3-*O*-{[*β*-glucopyranosyl(1→6)]-*O*-*β*-glucopyranosyl}-25-*O*-*β*-glucopyranoside (**3**), and 24(*R*)-stigmastan-3*β*,5*α*,6*β*-triol-25-ene 3-*O*-*β*-glucopyranoside (**4**), respectively. Their structures were elucidated by the combination of mass spectrometry (MS), one and two-dimensional NMR experiments and chemical reactions.

## 1. Introduction

**Figure 1:**
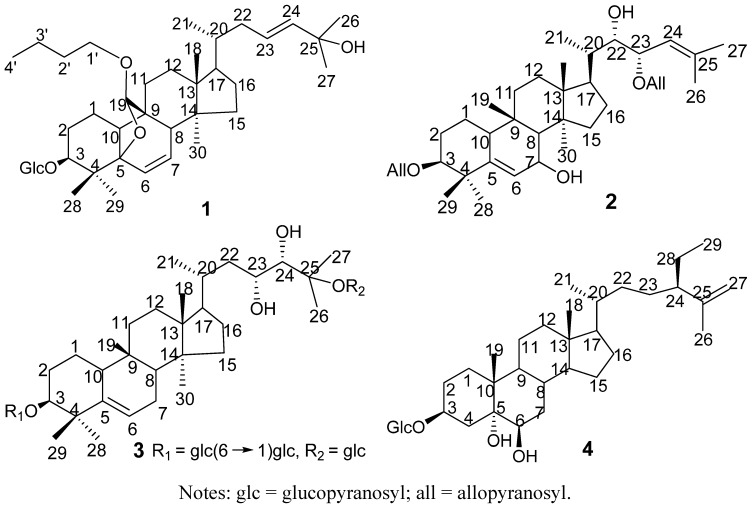
Structures of the new compounds from *M. charantia*.

The plant *Momordica charantia* L (cucurbitaceae) is cultivated in Asian countries. Its fruit, called kugua in Chinese and bitter melon in English, is a popular vegetable in Asian countries and is becoming a popular food supplement to lower blood glucose worldwide [1]. So far, more than 100 compounds, mainly cucurbitane- and oleanene-type triterpenes, have been isolated from the fruits, seeds, leaves, canes and roots of this genus. Recently, studies have discovered that the triterpenes from this genus showed biological activities such as antidiabetic [2], anti-HIV [3], anticancer [4] properties. Based on our continued interest in the discovery of sugar-reducing and anticancer compounds from *M. charantia*, we have examined the methanolic extracts of *M. charantia* from Chengjiang County, Yunnan Province, China. Herein, we report the isolation and structural elucidation of four new compounds from this plant (Figure 1). 

## 2. Results and Discussion 

In this report, we have examined the methanolic extract of the fruits of *M. charantia* and have isolated three new triterpenes **1–3** and one new steroidal glycoside **4**, together with ten known compounds: karaviloside II (**5**) [5], karaviloside III (**6**) [5], momordicoside K (**7**) [6], kuguaglycoside B (**8**) [7], momordicoside L (**9**) [8], momordicoside M (**10**) [9], momordicoside N (**11**) [9], momordicoside B (**12**) [10,11], momordicoside S (**13**) [2], and momordicoside A (**14**) [10,11], whose structures were determined by comparing their physical properties and the spectral data with those reported in the literature. 

**Figure 2:**
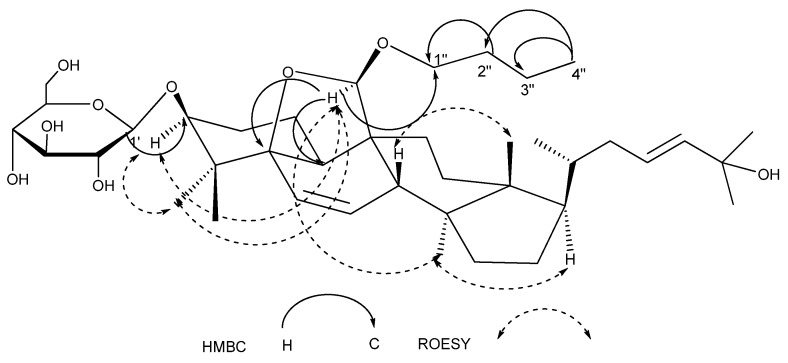
The Key HMBC and ROESY Correlations of Compound **1**.

Compound **1** was obtained as a white powder. The negative-ion quasimolecular ion peak was observed at *m/z* 689 [M-H]^−^, and the molecular formula C_40_H_66_O_9_ was determined by negative-ion HRESIMS measurement (*m/z* 689.4630 [M-H]^−^; calc. 689.4628). The ^1^H-NMR spectrum of **1** (Table 1 and Table 2) showed signals for six tertiary methyl groups at *δ* 0.85, 0.86, 0.91, 1.48, 1.55, and 1.55 (each 3H, s), a secondary methyl at *δ* 0.97 (3H, d, *J* = 5.1 Hz), a third methyl at 0.80 (3H, t, *J* = 7.2 Hz), and four olefinic protons at *δ* 6.15 (1H, d, *J* = 9.6 Hz), 5.63 (1H, dd, *J* = 9.6, 3.5 Hz), 5.94 (1H, *br. m*), and 5.94 (1H, *br. m*), as well as one anomeric proton at *δ* 4.96 (1H, d, *J* = 7.7 Hz). Acid hydrolysis of **1** furnished glucose, which was identified by TLC comparison with an authentic sample. In the ^13^C-NMR of **1** (Table 1 and Table 2), 30 aglycone carbon signals, six sugar signals and four *n*-butyl signals were found, indicating that **1** was a triterpene saponin. The ^13^C-NMR data of compound **1** were very similar to those of goyaglycoside-a [12], except that an OMe group at C-19 in goyaglycoside-a was replaced by an O-*n*-butyl group in **1**. In the ROESY experiment, the correlations between H-19 and H-3α, 3H-30α, 3H-28α were observed. As a result, H-19 was a α configuration. The above evidence and the analysis of HMBC, and ROESY data (Figure 2) confirmed that **1** was 19(*R*)-*n*-butanoxy-5*β*,19-epoxycucurbita-6,23-diene-3*β*,25-diol 3-*O*-*β*-glucopyranoside.

Compound **2** was obtained as a white powder. HRESIMS afforded C_42_H_69_O_14_ as a possible molecular formula of **2** ([M+Cl]^−^, m/z 797.4690, calc. 797.4687). The ^1^H-NMR spectrum of **2** (Table 1 and Table 2) showed signals for five tertiary methyl groups at *δ* 0.74, 0.86, 1.07, 1.35, and 1.54 (each 3H, s), a secondary methyl at *δ* 1.14 (1H, d, *J* = 6.6 Hz), two allylic methyls at 1.60, 1.80 (3H, s), and two olefinic protons at *δ* 6.08 (1H, d, *J* = 4.9 Hz), 5.51 (1H, d, *J* = 9.2 Hz), as well as two anomeric protons at *δ* 5.32 (1H, d, *J* = 7.8 Hz) and 5.61 (1H, d, *J* = 7.9 Hz). 

**Figure 3:**
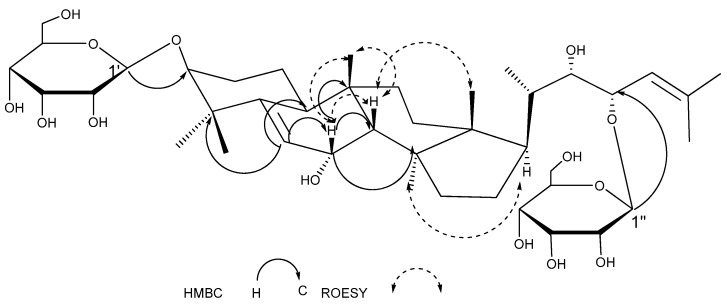
The Key HMBC and ROESY Correlations of Compound **2**.

The ^13^C-NMR of **2** (Table 1 and Table 2) showed 30 aglycone carbon signals and 12 signals from two sugars, which indicated that **2** was a triterpene saponin. Acid hydrolysis of **2** liberated allose, which was identified by TLC comparison with an authentic sample. The ^13^C-NMR data of **2** bore a resemblance to those of karaviloside V [5], with the exception of the signal of the hydroxy group at C-7 in **2** instead of an OMe group in karaviloside V. This deduction was in accordance with the evidence that C-7 in **2** was upshifted by 10.2 ppm when compared with C-7 in karaviloside V. Further analysis of the HMBC spectra of **2** proved the above deduction (Figure 3). The stereostructure of the aglycone moiety was characterized by ROESY experiment (Figure 3), which were observed between H-7 and 3H-19β, H-8β; H-8 and 3H-18β; 3H-30α and H-16α. Thus, the structure of **2** was confirmed as 23-*O*-*β*-allopyranosyle-cucurbita-5,24-dien-7*α*,3*β*,22(*R*),23(*S*)-tetraol 3-*O*-*β*-allopyranoside.

The molecular formula C_48_H_82_O_19_ of compound **3** was deduced by HRESIMS ([M-H]^−^ *m/z* 961.5366, calc. 961.5372). 1D-NMR data (Table 1 and Table 2) showed the presence of seven tertiary methyls, one secondary methyl, a trisubstituted double bond, six quaternary carbons, seven aglycone methylenes, and two sugar methylenes, as well as three anomeric carbons, which indicated that a triterpenoid glycoside with two sugar moieties. Acid hydrolysis of **3** released glucose, which was identified by TLC comparison with an authentic sample. Carbon signals from ^13^C-NMR data were superimposable on those of momordicoside S [2], except for those around the C-22 position. The signal of C-22 was upshifted by 29 ppm, the C-20, C-23 were upshifted by 10.5 ppm, 4.8 ppm, and the C-21, C-24 signals were downshifted by 3.6 ppm, 4.4 ppm, respectively, which suggested that a hydroxyl group attached to C-22 of **3** was disappeared compared with momordicoside S. The ^1^H-NMR and ^13^C-NMR spectra of C-20, 21, 22, 23, 24, and 25 of **3** were in good agreement with those of momordicoside R [2]. 

**Figure 4:**
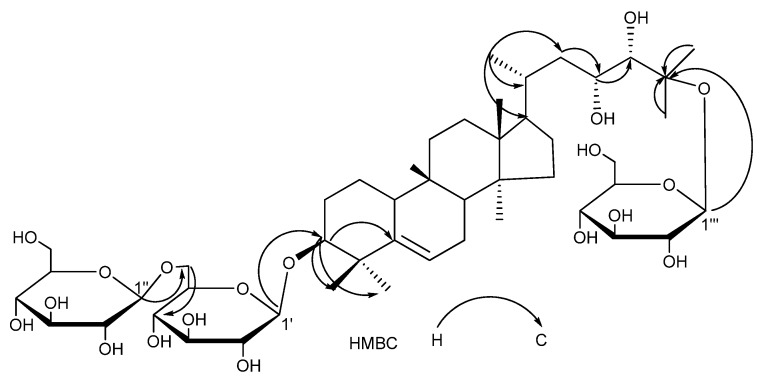
The Key HMBC Correlations of Compound **3**.

In the HMBC spectrum (Figure 4), long-range correlations were observed between H-1′ (*δ* 4.81) and C-3′ (*δ* 87.6), H-1′′ (*δ* 5.21) and C-6′ (*δ* 70.3), H-1′′′ (*δ* 5.30) and C-25 (δ 81.5), indicating that two glucoses were attached to C-3 and C-25 as well as the remain glucose was attached to C-6′ of the glucose linked to C-3 of aglycone. Based on the above results, compound **3** was identified as 23(*R*),24(*S*),25-trihydroxycucurbit-5-ene 3-*O*-{[*β*-glucopyranosyl(1→6)]-*O*-*β*-glucopyranosyl}-25-*O*-*β*-glucopyranoside.

**Figure 5:**
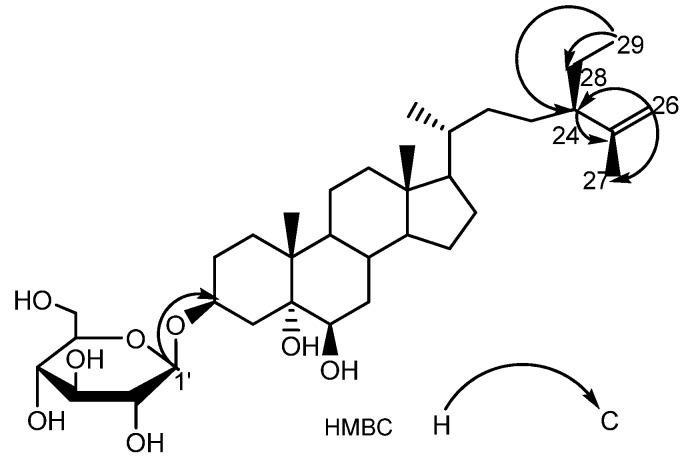
The Key HMBC Correlations of Compound **4**.

Compound **4** was obtained as a white powder. The molecular formula C_35_H_60_O_8_ was established by negative-ion HRESIMS measurement at m/z 643.3951 [M+Cl]^−^ (calc. 643.3976). The ^1^H-NMR spectrum of **4** (Table 3) showed signals for two tertiary methyl groups at *δ* 0.72 and 1.52 (each 3H, s), a secondary methyl at *δ* 0.96 (3H, d, *J* = 6.4 Hz), one allylic methyl group at 1.59 (3H, s), one third methyl at *δ* 0.83 (3H, t, *J* = 7.4 Hz) and two anomeric olefinic protons at *δ* 4.85 (1H, s), 4.78 (1H, d, *J =* 2.1 Hz) as well as one anomeric proton at δ 4.95 (1H, d, *J* = 7.7 Hz). The ^13^C-NMR data indicated that the presence of a steroidal glycoside bearing five methyls and a sugar unit. Acid hydrolysis of **4** afforded a glucose which was confirmed by TLC comparison with an authentic sample. All above data suggested that it was almost same as 24(*R*)-stigmastan-3*β*,5*α*,6*β*-triol 3-*O*-*β*-glucopuranoside [13], exception of a double bond between C-25 and C-26 in **4** instead of a methine group at C-25 and a methyl group at C-26. In the corresponding HMBC spectrum (Figure 5), the correlations between H-26 and C-24, 27 as well as between H-29 to C-24, 28 were observed. Thus, compound **4** was identified as 24(*R*)-stigmastan-3*β*,5*α*,6*β*-triol- 25-ene 3-*O*-*β*-glucopyranoside.

## 3. Experimental 

### 3.1. General 

Column chromatography (CC): silica gel (200–300 mesh; Qingdao Marine Chemical Products industry factory, China); Sephadex LH-20 (Pharmacia) and RP-18 silica gel (50–80 μm, Merck, Germany). TLC: silica gel G precoated plates (Qingdao Haiyan Chemical Co.) and Rp-18-F254S precoated plates (Merck, Germany); Spots were visualized by spraying with 10% aq. H_2_SO_4_ soln., followed by heating. Optical rotations: Horiba SEAP-3000 spectropolarimeter. IR spectra: Shimadzu IR-450 instrument, with KBr pellets; in cm^−1^. NMR Spectra: Bruker AC-400 (The ^13^C-NMR was measured in Bruker AC-400) or DRX-500 (The ^1^H-NMR was measured in DRX-500) instruments; chemical shifts δ in ppm rel. to SiMe_4_, coupling constants *J* in Hz. FAB-MS (negative-ion mode; glycerol matrix) and HR-ESI-MS: VG-Auto-Spec-3000 and Thermo-Finnigan LCQ-Advantage spectrometer; in m/z (rel. int. in % of the base peak).

### 3.2. Plant Material

The fresh fruits of *M. charantia* were purchased from in Chengjiang County, Yuxi City, Yunnan Province, P.R. China, in August 2008, and identified by Prof. Shukun Chen.

### 3.3. Extraction and Isolation 

Dried and powdered fruits of *M. charantia* (35 kg) were extracted with MeOH at 70 °C (4 × 50 L). Removal of solvent under vacuum gave the MeOH extract (700 g), which was suspended in water and partitioned with petroleum ether, ethyl acetate, and *n*-BuOH. The *n*-BuOH layer was concentrated and the residue (300 g) was chromatographed on an D_101_ resin column, eluted with water, MeOH, (Me)_2_CO. The MeOH residue were fractionated by normal phase column chromatography, which was eluted with gradient CHCl_3_/MeOH (30:1, 10:1, 5:1, 3:1, 1:1, 0:1) and afforded six fractions (Fr.1–Fr.6). Fr.2 was separated on normal phase silica gel eluted with CHCl_3_/MeOH (20:1, 10:1) and CHCl_3_/(Me)_2_CO (3:1, 1.5:1,1:2) repeatedly, then on *RP-18* silica gel eluted with MeOH/H_2_O (60%–80%), finally yielded compound **1** (8 mg), **5** (11 mg), **6** (24 mg); Fr.3 was purified by a chromatographic column on normal phase silica gel eluted with CHCl_3_/MeOH (8:1, 5:1) and on reversed phase silica gel eluted with MeOH/ H_2_O (50%–70%) to yield compound **4** (15 mg), **7** (156 mg), **8** (13 mg), **9** (13 mg), **10** (29 mg); Fr.4 was separated on a normal phase silica gel column eluted with CHCl_3_/MeOH (5:1, 3:1) and on RP-18 silica gel column eluted with 45% of MeOH/ H_2_O, then on Sephadex LH-20 column (MeOH) to afford compound **2** (10 mg), **11** (30 mg); Compound **3** (10 mg), **12** (30 mg), **13** (15 mg), **14** (250 mg) were isolated from Fr.5 by using the chromatographic column on normal phase silica gel column eluted with *n*-BuOH/AcOEt/ H_2_O (4:4:1 upper layer) and on reversed phase silica gel column eluted with MeOH/ H_2_O (45%) as well as on Sephadex LH-20 column (MeOH).

### 3.4. Acid Hydrolysis of Compounds ***1**–**4***

Compounds **1**–**4** (5 mg each) in 1 N HCl-CH_3_OH (1:1, 2 mL) were each heated at 90 °C for 4 h in a water bath. The reaction mixtures were neutralized with AgCO_3_, filtered, and then extracted with CHCl_3_ (2 mL × 3). After concentration, each H_2_O layer (monosaccharide portion) was examined by TLC with BuOH/acetic ether/H_2_O (4:1:5 upper layer) and compared with authentic samples (allopyranosyl and glucopyranosyl), Rf_all_ = 0.33, Rf_glc_ = 0.36.

### 3.5. Spectral Data

*19(R)-n-Butanoxy-5β,19-epoxycucurbita-6,23-diene-3β,25-diol* *3-O-β-glucopyranoside* (**1**). C_40_H_66_O_9_, white powder; ${\left[\alpha \right]}_{D}^{25}$ = −79.0° (c 0.11 MeOH); negative FABMS: *m/z* 689 [M−H]^−^; HRESIMS: [M-H]^−^ m/z 689.4630 (cal. 689.4628); IR (KBr) ν_max_: 3413, 2930, 1633, 1453, 1375, 1073 cm^−1^; ^1^H-NMR (C_5_D_5_N, 500 MHz) and ^13^C-NMR (C_5_D_5_N, 125 MHz): see Table 1, Table 2.

*23-O-β-Allopyranosyle-cucurbita-5,24-dien-7α,3β,22(R),23(S)-tetraol-3-O-β-allopyranoside* (**2**). C_42_H_69_O_14_, white powder; ${\left[\alpha \right]}_{D}^{25}$ = −1.4° (c 0.1 MeOH); negative FABMS: *m/z* 797 [M−H]^−^; HRESIMS: [M−H]^−^ m/z 797.4690 (cal. 797.4687); IR (KBr) ν_max_: 3404, 2930, 1642, 1453, 1372, 1071, 970 cm^−^^1^; ^1^H-NMR (C_5_D_5_N, 500 MHz) and ^13^C-NMR (C_5_D_5_N, 125 MHz): see Table 1, Table 2.

*23(R),24(S),25-Trihydroxycucurbita-5-ene-3-O-{[β-glucopyranosyl(1→6)]-O-β-glucopyranosyl}-25-O-β-glucopyranoside* (**3**). C_48_H_82_O_19_, white powder; ${\left[\alpha \right]}_{D}^{25}$ = +1.1° (c 0.09 MeOH); negative FABMS: *m/z* 961 [M−H]^−^; HRESIMS: [M-H]^−^ m/z 961.5366 (cal. 961.5372); IR (KBr) ν_max_: 3400, 2926, 1648, 1455, 1377, 1072, 985 cm^−^^1^; ^1^H-NMR (C_5_D_5_N, 500 MHz) and ^13^C-NMR (C_5_D_5_N, 125 MHz): see Table 1, Table 2.

*24(R)-Stigmastan-3β,5α,6β-triol-25-ene 3-O-β-glucopyranoside* (**4**). C_35_H_60_O_8_, white powder; ${\left[\alpha \right]}_{D}^{25}$ = −50.2° (c 0.1 MeOH); negative FABMS: *m/z* 607 [M−H]^−^; HRESIMS: [M+Cl]^−^ m/z 643.3951 (cal. 643.3976); IR (KBr) ν_max_: 3400, 2920, 1640, 1450, 1370, 1070, 980 cm^−1^; ^1^H-NMR (C_5_D_5_N, 500 MHz) and ^13^C-NMR (C_5_D_5_N, 125 MHz): see Table 3.

## 4. Conclusion

We have isolated three new triterpenes and one new steroidal glycoside from the fruits of *M. charantia*, together with ten known compounds karaviloside II, karaviloside III, momordicoside K, kuguaglycoside B, momordicoside L, momordicoside M, momordicoside N, momordicoside B, momordicoside S, and momordicoside A. Although we researched on the same specie from different localities [3,7], the chemical constitutions of them were different. So, for better using the fruits of *M. charantia* as a food supplement or a plant extract to lower blood glucose, we had better pay attention to their chemical constitutions in different localities. 

## Figures and Tables

**Table 1:** ^1^H- and ^13^C-NMR data for the aglycone moieties of **1**, **2**, **3** (pyridine-*d_5_*, *δ* in ppm and *J* in Hz).

Atom No.	**1**		**2**		**3**	
	σ_C_	σ_H_	σ_C_	σ_H_	σ_C_	σ_H_
1	19.6	1.30~1.33 (1H, m)	22.8	1.97~2.00,1.52~1.55 (2H, 2m)	22.8	1.80~1.84,1.60~1.58 (2H, 2m)
2	27.4	2.20~2.24, 1.72~1.76 (2H, 2m)	28.8	2.45~2.49 (2H, m)	29.1	2.58 (1H, dd, *J* = 10.9, 3.8)
3	83.7	3.73, 1H, br s	87.9	3.63, 1H, br s	87.6	3.68, 1H, br s
4	39.2		41.9		41.8	
5	85.4		146.5		143.3	
6	133.2	6.15 (1H, d, *J* = 9.6)	122.5	6.08 (1H,d, *J* = 4.9 )	118.8	5.46 (1H, d, *J* = 5.4)
7	131.6	5.63 (1H, dd, *J* = 9.6, 3.5)	67.4	4.23~4.27 (1H, m)	24.5	1.65~1.68,1.70~1.74 ( 2H, 2m)
8	42.3	3.10~3.15 (1H, m)	53.8	2.27, 1H, s	43.8	1.61, 1H (overlap)
9	48.1		34.6		34.7	
10	41.6	2.43 (1H, dd, *J* = 12.3, 5.5)	40.9	2.10~2.12 (1H, m)	38.6	2.23 (1H, overlap)
11	23.3	1.60~1.65, 1.71~1.76 (2H, 2m)	33.1	1.45~1.50,1.40~1.43 (2H, 2m)	32.5	1.29~1.32,1.50~1.55 (2H, 2m)
12	30.9	1.47~1.51, 1.59~1.63 (2H, 2m)	30.7	1.52~1.57,1.41~1.46 (2H, 2m)	30.9	1.40~1.45,1.50~1.55 (2H, 2m)
13	45.3		46.5		46.6	
14	48.3		48.4		49.6	
15	33.9	1.20~1.29 (2H, m)	35.2	1.32~1.36 (2H, m)	35.0	1.14 (1H, overlap)
16	28.2	1.85~1.93, 1.25~1.29 (2H, 2m)	27.9	1.85~1.88 (2H, m)	28.5	1.90~1.96,1.40~1.45 (2H, 2m)
17	50.4	1.46~1.52 (1H, m)	46.5	1.80~1.84 (1H, m)	51.9	1.60 (1H, overlap)
18	14.7	0.91, 3H, s	15.1	0.86, 3H, s	15.7	0.84, 3H, s
19	111.3	4.95, 1H, s	30.0	1.35, 3H, s	28.3	0.85, 3H, s
20	36.7	1.49~1.53 (1H, m)	39.3	2.32~2.36 (2H, m)	32.7	2.19~2.23 (1H, m)
21	18.9	0.97 (3H, d, *J* = 5.1)	14.9	1.14 (1H, d, J = 6.6)	19.2	1.15 (1H, d, *J* = 6.2)
22	39.6	2.19~2.24, 1.79~1.85 (2H, 2m)	76.8	3.98~4.04 (1H, m)	42.9	1.10~1.14,2.00~2.03 (2H, 2m)
23	124.5	7.21 (1H, br s)	81.3	4.60~4.64 (1H, m)	69.7	4.29~4.35 (1H, m)
24	141.8	5.94 (1H, d, *J* = 8.7)	124.7	5.51 (1H, d, *J* = 9.2)	78.6	4.26 (1H,overlap)
25	69.7		135.4		81.5	
26	30.9	1.55. 3H, s	26.3	1.60, 3H, s	23.5	1.89, 3H, s
27	30.8	1.55. 3H, s	18.7	1.80, 3H, s	24.6	1.78, 3H, s
28	21.3	1.48, 3H, s	28.8	1.07, 3H, s	28.3	1.04, 3H, s
29	24.9	0.85, 3H, s	26.0	1.54, 3H, s	26.0	1.49, 3H, s
30	20.0	0.86, 3H, s	18.1	0.74, 3H, s	18.0	0.78, 3H, s

**Table 2:** ^1^H and ^13^C NMR data for substituents of **1**, **2**, **3** (in pyridine-*d_5_*, *δ* in ppm and *J* in Hz).

	**1**		**2**			**3**		
Atom No.	σ_C_	σ_H_	Atom No.	σ_C_	σ_H_	Atom No.	σ_C_	σ_H_
Glc-1′	105.3	4.96 (1H, d, *J* = 7.7)	All-1′	104.9	5.32 (1H, d, *J* = 7.8)	Glc-1′	107.1	4.81 (1H, d, *J* = 7.7)
2′	76.3	4.06 (1H, t, *J* = 8.3)	2′	72.3	3.90 (1H, dd, *J* = 7.6, 2.7)	2′	75.5	3.83, 1H
3′	78.0	4.20~4.26 (1H, m)	3′	73.5	4.60~4.67 (1H, m)	3′	78.5	3.85, 1H
4′	71.9	4.17~4.21 (1H, m)	4′	69.3	4.12~4.17 (1H, m)	4′	71.8	4.03, 1H
5′	78.8	3.97~4.00 (1H, m)	5′	76.1	4.40~4.44 (1H, m)	5′	77.5	4.02, 1H
6′	63.0	4.55~4.60, 4.37~4.41 (2H, 2m)	6′	63.3	4.46~4.51, 4.32~4.37 (2H, 2m)	6′	70.3	4.27~4.31,4.79~4.85 (2H, 2m)
*n*-butyl-1′′	70.0	3.90~3.95,3.38~3.41 (2H, 2m)	All-1′′	103.7	5.61 (1H, d, J = 7.9)	Glc-1′′	105.4	5.21 (1H, d, *J* = 7.7)
2′′	32.2	1.40~1.42 (2H, m)	2′′	73.2	4.00~4.04 (1H, m)	2′′	75.4	3.92~3.97 (1H, m)
3′′	18.8	1.85~1.88, 1.40~1.42, (2H, 2m)	3′′	73.1	4.69~4.72 (1H, m)	3′′	78.6	3.86~3.90 (1H, m)
4′′	14.0	0.80 (3H, t, *J* = 7.2)	4′′	69.0	4.20~4.23 (1H, m)	4′′	71.8	4.12~4.17 (1H, m)
			5′′	75.7	4.42~4.45 (1H, m)	5′′	79.1	4.15~4.19 (1H, m)
			6′′	63.0	4.44~4.48, 4.30~4.32 (2H, 2m)	6′′	62.8	4.29~4.31,4.47~4.51 (2H, 2m)
						Glc-1′′′	97.9	5.30 (1H, d, *J* = 7.8)
						2′′′	75.3	3.97~4.00 (1H, m)
						3′′′	78.7	3.73~3.79(1H, m)
						4′′′	71.7	3.86~3.91(1H, *m*)
						5′′′	79.1	4.00~4.10 (1H, *m*)
						6′′′	62.8	4.30~4.35,4.50~4.55 (2H, 2*m*)

**Table 3:** ^1^H and ^13^C NMR of compound **4** (in pyridine-*d_5_*, *δ* in ppm and *J* in Hz).

Atom No.	σ_C_	σ_H_	Atom No.	σ_C_	σ_H_
1	33.1	2.01~2.09,1.40~1.47(2H, 2m)	19	17.0	1.52, 3H, s
2	29.9	2.18~2.24,1.95~1.99(2H, 2m)	20	35.9	1.38~1.44 (1H, m)
3	75.0	4.90~4.98 (1H, m)	21	18.9	0.96 (3H, d, *J* = 6.4)
4	38.7	2.76~2.83,2.44~2.49(2H, 2m)	22	34.0	1.29~1.34,0.96~1.00 (2H, 2m)
5	75.5		23	26.8	1.29~1.34 (2H, m)
6	76.3	4.10~4.15 (1H, m)	24	49.8	1.81~1.88 (1H, m)
7	35.7	2.17~2.22,1.89~1.93(2H, 2m)	25	147.7	
8	31.2	2.11~2.16 (1H, m)	26	112.0	4.85 (1H, s), 4.78 (1H, d, *J =* 2.1)
9	45.8	1.86~1.91 (1H, m)	27	17.8	1.59, 3H, s
10	39.2		28	29.8	1.31~1.38, 1.19~1.25 (2H, 2m)
11	21.7	1.43~1.50,1.40~1.46(2H, 2m)	29	12.4	0.83 (3H, t, *J* = 7.4)
12	40.6	2.00~2.03,1.15~1.19(2H, 2m)	Glu-1′	102.3	4.95 (1H, d, *J* = 7.7)
13	43.1		2′	75.3	4.04 (1H, t, *J* = 8.3)
14	56.6	1.17~1.18 (1H, m)	3′	78.6	3.70~3.74 (1H, m)
15	24.7	1.59~1.63,1.05~1.10(2H, 2m)	4′	71.5	4.21~4.25 (1H, m)
16	28.6	1.72~1.79,1.19~1.22(2H, 2m)	5′	78.3	4.10~4.17 (1H, m)
17	56.5	1.02~1.07 (1H, m)	6′	62.7	4.40~4.47,4.31~4.36 (2H, 2m)
18	12.3	0.72, 3H, s			
